# Systemic Chemotherapy for Progression of Brain Metastases in Extensive-Stage Small Cell Lung Cancer

**DOI:** 10.1155/2015/620582

**Published:** 2015-08-26

**Authors:** Nagla Abdel Karim, Ananta Bhatt, Lauren Chiec, Richard Curry

**Affiliations:** ^1^The Vontz Center for Molecular Studies, Cincinnati, OH 45267, USA; ^2^Department of Internal Medicine, University of Cincinnati Medical Center, Cincinnati, OH 45219, USA; ^3^University of Cincinnati Medical Center, Cincinnati, OH 45219, USA; ^4^Department of Neurology and Rehabilitation Medicine, University of Cincinnati Medical Center, Cincinnati, OH 45219, USA

## Abstract

Lung cancer is the most common cause of cancer related mortality in men and women. Approximately 15% of lung cancers are small cell type. Chemotherapy and radiation are the mainstay treatments. Currently, the standard chemotherapy regimen includes platinum/etoposide. For extensive small cell lung cancer, irinotecan and cisplatin have also been used. Patients with relapsed small cell lung cancer have a very poor prognosis, and the morbidity increases with brain metastases. Approximately 10%–14% of small cell lung cancer patients exhibit brain metastases at the time of diagnosis, which increases to 50%–80% as the disease progresses. Mean survival with brain metastases is reported to be less than six months, thus calling for improved regimens. Here we present a case series of patients treated with irinotecan for progressive brain metastases in small cell lung cancer, which serves as a reminder of the role of systemic chemotherapy in this setting.

## 1. Introduction

Lung cancer is the most common cause of cancer related deaths. Approximately 15% of lung cancers are small cell lung cancer (SCLC), which is an aggressive cancer with rapid growth and early development of widespread metastases [1–3]. Around 60–70% of patients present with extensive-stage disease.

SCLC is initially very sensitive to chemotherapy. In patients with limited stage disease, the goal of treatment is curative utilizing cisplatin/etoposide [4]. Although SCLC is responsive to upfront treatment, most patients relapse with relatively resistant disease [5, 6]. 60%–90% of patients with limited stage disease respond to first-line treatment, and 40–50% achieve a complete response [7]. However, due to relapses, the median survival ranges from 12 to 20 months, with only 6–12% living more than 5 years. Patients with extensive-stage disease have lower overall response and shorter survival rates, with median survival between 7 and 11 months and 2-year survival in less than 5% [7].

Patients with relapsed or progressive SCLC have a poor prognosis. Median survival is only 4 to 5 months when treated with second-line chemotherapy. Recently, combination of irinotecan and carboplatin was added to NCCN guidelines as an option for patients with extensive-stage disease. Single agent topotecan is FDA-approved for treatment of SCLC patients who had an initial response to chemotherapy but progressed after 2 to 3 months [8–11]. Both topotecan and irinotecan have shown to be effective in treating patients with brain metastases [12].

We further demonstrate the effects of irinotecan through a case series of four patients with SCLC and progressive brain metastases who had exhausted standard treatment options with systemic chemotherapy and cranial irradiation.

## 2. Case  1

**Figure 1:**
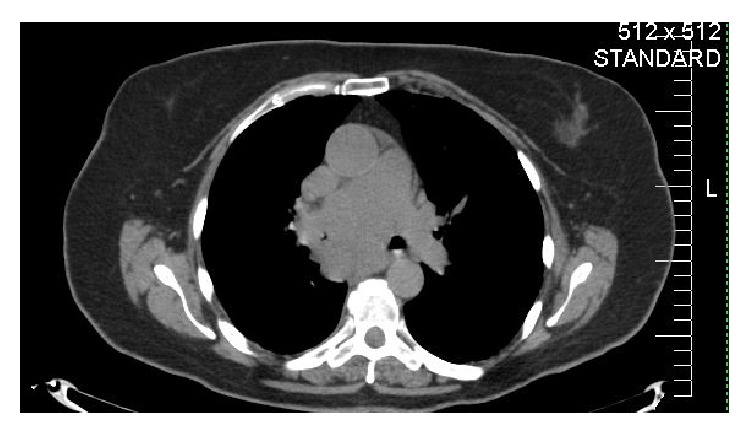
CT of right lung mass occluding the bronchus intermedius and a subcarinal mass which invades the left main bronchus.

Initial computed tomography (CT) chest scan performed on a 70-year-old female showed a mass in the right lung occluding the bronchus intermedius and a subcarinal mass consistent with metastatic adenopathy (Figure 1), which was found to be SCLC. Magnetic resonance imaging (MRI) of the brain showed no evidence of metastases. She received four cycles of cisplatin/etoposide plus chest radiation, followed by PCI.

**Figure 2:**
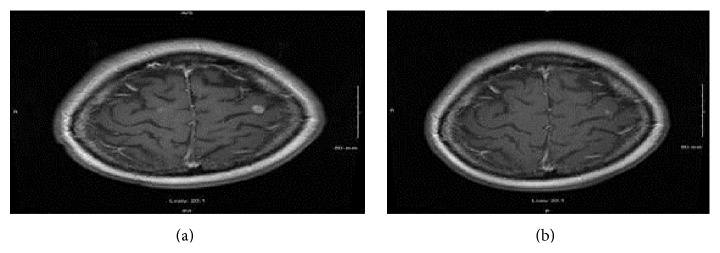
(a, b) Brain metastasis regression after 4 cycles of irinotecan: (a) prechemotherapy and (b) postchemotherapy.

Five months later, PET/CT showed increased fludeoxyglucose (FDG) activity in a right lower lobe nodule, consistent with residual tumor. There was increased FDG uptake in the right chest wall, left supraclavicular region, retroperitoneum, adrenal glands, bony pelvis, and left lobe thyroid nodule. MRI of the brain showed numerous enhancing foci consistent with metastatic disease (Figure 2(a)). She began second-line chemotherapy with irinotecan: 100 mg/m^2^ on days 1, 8, and 15 of a 21-day cycle.

The patient showed an excellent response for an additional 14 months before repeat MRI of the brain demonstrated progression of disease.

## 3. Case  2

A 61-year-old female underwent MRI of the head which showed a large, enhancing, extra-axial mass lesion within the right posterior fossa, which was ultimately diagnosed as metastatic small cell carcinoma. CT of chest, abdomen, and pelvis revealed a soft tissue nodule in the left upper lobe of the lung with adjacent hilar lymphadenopathy.

The patient underwent PCI followed by 4 cycles of platinum/etoposide with a near-complete response. Five months later, MRI of the head demonstrated extensive subependymal metastatic disease extending into the internal auditory canals and evolving subarachnoid dissemination of disease. CT of the chest showed recurrent tumor in the left upper lobe. She was started on irinotecan at 100 mg/m^2^. The patient continued to tolerate maintenance irinotecan for 8 months, when she began to experience increased diarrhea and weakness and irinotecan was held. Two months later, MRI of the head and CT of the chest demonstrated stable disease. The patient then began to decline functionally and chose to forgo further follow-up.

## 4. Case  3

A 57-year-old man underwent MRI of the brain which demonstrated a left posterior frontal hematoma with mild enhancement, an enhancing soft tissue nodule along the lateral wall of the right lateral ventricle, and an enhancing lesion in the left occipital lobe, all consistent with metastatic disease. He underwent a left posterior temporal craniotomy, with no residual tumor on MRI. Biopsy was nondiagnostic. CT of the chest showed a 5.4 × 3.8 cm soft tissue mass in the right lower lobe of the lung with right hilar and paratracheal lymphadenopathy, consistent with primary lung carcinoma.

MRI of the head two months later showed new peripheral enhancement surrounding the previous surgical cavity, consistent with recurrent or residual tumor. It also showed mild increase in size of the right lateral ventricle lesion and an increase in size of the left occipital lobe lesion. EBUS-FNA demonstrated small cell carcinoma.

He then underwent palliative whole brain radiotherapy followed by systemic chemotherapy with platinum/etoposide for a total of 6 cycles. CT of the chest six months later showed stable disease.

Two months later, CT of the chest showed progression of disease and MRI of the head showed worsening metastatic disease. The patient was started on irinotecan at 100 mg/m^2^. CT of the chest after cycle 3 demonstrated a decrease in the right lower lobe mass, with stable to slightly decreased paratracheal and hilar lymphadenopathy. There was an increase in mediastinal adenopathy and the left lower lobe pulmonary nodule. MRI of the head after cycle 5 showed a mixed response, with a variable decrease in size of the previously demonstrated lesions as well as multiple new punctate lesions all measuring less than 5 mm. The patient continued on weekly irinotecan 5 months after initiation.

## 5. Case  4

A 58-year-old male was found to have several 3-4 cm masses located bilaterally at zone 2 of the neck, as well as a mass in the left posterior axillary chain. CT of the neck showed extensive bilateral neck lymphadenopathy. CT of the chest demonstrated a lobulated left hilar mass extending into the aortic-pulmonic window measuring 7.4 × 6 cm. Additionally, enlarged left hilar, subcarinal, and prevascular lymph nodes as well as subcentimeter nodular opacities in the left upper lobe were seen. Multiple low-density lesions were seen within the liver and spleen. Biopsy of the left axillary mass showed small cell carcinoma. MRI of the head demonstrated innumerable small enhancing lesions consistent with metastatic disease.

The patient began systemic chemotherapy with platinum/etoposide for 4 cycles. Four months later, the patient had clinical PD, with an increase in painful submandibular lymphadenopathy. He received palliative radiation to his cervical nodes, humerus, and bilateral sacroiliac joints. He was started on irinotecan at 100 mg/m^2^ and completed 2 cycles. Progression of his systemic disease was then seen on CT pulmonary angiography, and new cervical, thoracic, and lumbar metastases were seen on MRI of the spine. He received palliative radiation to his spine. However, MRI of the head two months after initiating irinotecan showed no signs of metastatic disease.

## 6. Discussion

SCLC is associated with a poor prognosis. Many studies utilizing systemic chemotherapy have shown improved response and survival rates in SCLC patients with recurrent disease and brain metastases. Our patients were treated with second-line irinotecan, which they tolerated well, and showed remarkable responses, especially with regard to brain metastases. This translates into significant improvement in quality of life and survival.

While studies have demonstrated excellent response rates with systemic chemotherapy in patients with relapsed SCLC and brain metastases (single agent camptothecin analogues in our case series), further studies are needed to better understand the role. The ideal agent, timing, dose, and frequency are unclear. The relationship with cranial irradiation is of great interest.

As evidenced in our cases, patients who have brain metastases after radiation can still benefit from systemic chemotherapy. A valid question is whether these agents can be administered prior to radiation, namely, in cases of a small number of asymptomatic metastases, although the accepted practice for SCLC remains to administer PCI initially.

The first two patients described in our case series had a benefit of one-year survival with continued irinotecan therapy after their diagnosis of uncontrolled brain metastases. The third patient is still undergoing treatment and has been tolerating it well for five months. Although the fourth patient rapidly progressed systemically, he had a near-complete response of his brain metastases.

## 7. Conclusion

Advances in the treatment of SCLC with brain metastases are clearly needed. These patients have few treatment options due to chemotherapy resistance and limited availability of agents that cross the BBB. In multiple preclinical and clinical studies, topotecan/irinotecan has shown to be effective as second-line monotherapy for treatment of brain metastases. Therefore, systemic chemotherapy remains an option for treatment after progression on first-line chemotherapy and radiation.
